# Exploring the Historical Background and Clinical Implications of Electrocardiogram in the Context of Chagas Disease Research

**DOI:** 10.1590/0037-8682-0506-2023

**Published:** 2023-12-15

**Authors:** Alejandro Marcel Hasslocher-Moreno, Roberto Magalhães Saraiva, Telêmaco Luiz da Silva, Sergio Salles Xavier, Andréa Silvestre de Sousa

**Affiliations:** 1 Instituto Nacional de Infectologia Evandro Chagas, Fundação Oswaldo Cruz, Rio de Janeiro, RJ, Brasil. Instituto Nacional de Infectologia Evandro Chagas Fundação Oswaldo Cruz Rio de Janeiro RJ Brasil; 2 Cardion - Cardiologia Preventiva e Avançada, Uberlândia, MG, Brasil. Cardion - Cardiologia Preventiva e Avançada Uberlândia MG Brasil; 3 Universidade Federal do Rio de Janeiro, Faculdade de Medicina, Rio de Janeiro, RJ, Brasil. Universidade Federal do Rio de Janeiro Faculdade de Medicina Rio de Janeiro RJ Brasil

**Keywords:** Chagas disease, Chagas cardiomyopathy, Electrocardiogram, Standardization

## Abstract

Chagas disease (CD) remains one of the most significant endemic diseases in Latin America. Approximately 30% of individuals with CD develop the cardiac form, the main determinant of morbidity and mortality, which is characterized by typical electrocardiogram (ECG) changes caused by chronic chagasic cardiopathy (CCC). This review accentuates to how crucial it is for research teams and reference centers that treat patients with CD to standardize ECG in CCC. This was a non-systematic review of the literature. ECG is the most widely used examination in the diagnosis and evaluation of CCC, and it is also employed in epidemiological surveys, risk stratification for cardiovascular events and death, and monitoring the clinical progression of the disease. Carlos Chagas and Eurico Villela published the first work addressing CCC in 1922. Other works followed, including the study by Evandro Chagas' which was the first to perform ECG in CD, culminating in Francisco Laranja's seminal work in 1956. Since the 1980s, standardizations and ECG reading codes for CD have been established. This standardization aimed to code complex arrhythmias and characteristic ventricular conduction disorders and standardize ECG readings for clinical and epidemiological studies in CD. Nearly all existing electrocardiographic abnormalities can be found in CD, with a predominance of abnormalities in the formation and conduction of cardiac stimuli. The complex and heterogeneous substrate of CD with varied electrocardiographic manifestations poses a significant challenge when comparing studies involving patients with CCC, emphasizing the need for ECG standardization in CD.

## INTRODUCTION

More than one hundred years after Chagas disease (CD) was first described, it remains a major public health problem in most Latin American countries^1^. If the individual is not treated with trypanocidal drugs in the disease’s acute phase, the disease progresses to the chronic phase. In this condition, most patients remain in the indeterminate form, with no clinical, electrocardiographic, or radiological changes. However, approximately 30% of individuals develop the cardiac form, the main determinant of morbidity and mortality, which is characterized by typical electrocardiogram (ECG) changes caused by chronic chagasic cardiopathy (CCC)^2^.

Because of its practicality, low cost, and good sensitivity to detect, quantify, and monitor most manifestations of CCC, ECG examination has been very valuable for the study of morbidity and mortality in CD^3^. It holds significant epidemiological value, being the method of choice for disease stratification in population-based, cross-sectional, and longitudinal studies in endemic areas^4^. Electrocardiographic changes usually precede the appearance of symptoms and abnormalities revealed by physical examination and radiological study of the heart^5^. Moreover, the ECG holds prognostic significance, as it is established that the sooner electrocardiographic alterations manifest, the bleaker the outlook becomes^6^^,^^7^.

The prevalence of electrocardiographic changes in patients with CD is higher in males and can be approximately nine times greater than that in seronegative controls, especially in patients aged between 25 and 44 years old^8^. In older age groups, especially after 60 years of age, the prevalence of electrocardiographic changes decreases compared with controls, mainly because of the increased frequency of electrocardiographic changes in seronegative individuals due to aging. Nevertheless, CD has a prognostic impact, indicating the progressive nature of the disease^9^. During the disease course, the ECG shows progressive changes that reflect the worsening of myocardial damage resulting from chronically active diffuse myocarditis. ECG changes occur in an increasing and progressive manner, starting with borderline alterations and progressing to more complex changes as the myocardial function deteriorates. These changes are more frequent and severe in patients with advanced Chagas heart disease and indicate a worse prognosis^3^.

We conducted a simple non-systematic review of the literature using the PubMed and SciELO databases and search for all available references published until July 2023. We also searched relevant gray literature from international and governmental organizations, including the World Health Organization and bulletins from the Brazilian Ministry of Health. The search terms included: Chagas or Chagas heart disease; and electrocardiogram or standardization.

The aim of this review was to address the historical aspects of ECG use in CD and highlight the importance of interpreting ECG changes to define the diagnosis of CCC. Using standardized ECG criteria for CCC diagnosis, previously defined by national and international guidelines, is essential for reference institutions that attend to patients with CD and for research groups following cohorts in cross-sectional and longitudinal studies.

## HISTORY

In his seminal work, Carlos Chagas, described, the acute phase of CD^10^. Carlos Chagas, in partnership with Eurico Villela, also identified and described the chronic phase of the disease, which is characterized by cardiopathy^11^. In this first clinical study on the cardiac form of CD, the authors focused the frequency and intensity of cardiac rhythm disorders during the chronic phase of the disease. They highlighted the importance of conduction and stimulus formation disturbances, pointing out the frequency of ventricular extrasystoles and the even greater severity of prognosis if atrial fibrillation or flutter were to occur^12^. Based on this work, other studies established the use of ECG in CCC. Vilella and Bicalho performed the first ECG recordings of CCC in five clinically evaluated patients, all of whom presented with ventricular extrasystole^13^. Evandro Chagas, the son of Carlos Chagas, updated the cardiac form of CD in its etiological, anatomical, clinical, electrocardiographic, and therapeutic aspects^14^. Using ECG, Evandro Chagas systematized the “irregularities of cardiac activity according to alterations of the fundamental properties of the myocardium” presenting: “total tachycardia”; “total bradycardia”; “total arrhythmia”; “nodal rhythm”; “idioventricular rhythm”; “sino-atrial block”; “intra-atrial block”; “intraventricular block”; “partial block”; “total block”; “sinus extrasystole”; “nodal extrasystole”; “ventricular extrasystoles and grouped extrasystoles (bigeminy, trigeminy, and paroxysmal tachycardia)”; “atrial and ventricular tachysystole”; “atrial and ventricular fibrillation”; “alternance”; “galloping rhythm”; and “T wave changes”. Two years later, in a second study, Evandro Chagas performed ECG and chest radiography on four patients with CD, detailing the most prominent electrocardiographic changes in each patient: in the first patient it was 2nd-degree 2:1 atrioventricular block, polymorphic ventricular extrasystole in the second, frequent monomorphic ventricular extrasystole in the third, and 2nd-degree sinoatrial block in the fourth patient. These early electrocardiographic findings reflect the arrhythmogenic pattern, characteristics and frequent conduction disturbances of CCC^15^. 

Dias et al. identified a 50% prevalence of ECG abnormalities in a series of cases of CD studied in the city of Bambuí, with a predominance in adolescents and young individuals between 11 and 20 years old. They also observed that ECG changes, usually related to a dismal prognosis, occurred in asymptomatic individuals and were associated with a high incidence of sudden death in this population^16^. Magalhães and Freire commented on the “improvement in the interpreting of electrocardiographic tracings” that would allow recording of seemingly unknown aspects. They concluded that “introduction of multiple precordial leads will be crucial regarding the form of the electrocardiogram”^17^. 

Two other studies on CD were published, both of which used ECG to assess and study CCC. In an extensive review of the disease, Laranja et al. commented that “the electrocardiographic method plays a relevant role in diagnosing this cardiopathy”. They added that “the electrocardiographic picture of the chronic cardiopathy of Chagas disease, recently described, constitutes one of the most diverse and curious findings in cardiology, representing almost all types of ECG alterations”. They also described the main ECG changes, highlighting “the ventricular extrasystoles, right bundle branch block, atrioventricular blocks of all degrees, and primary T wave changes” as particularly frequent. They were also the first authors to report a high incidence of right bundle branch blocks^18^. Rodovalho et al. gathered 80 cases from various clinical services at the Hospital das Clínicas of the School of Medicine at the University of São Paulo. Among the alterations found, conduction abnormalities predominated at 71.2% and were mostly represented by a right bundle branch block, followed by rhythm changes at 62.5%, with ventricular extrasystoles being the main finding. The authors concluded that “Conduction abnormalities are particularly notable and seem to prevail in chagasic cardiopathy when compared to other types of cardiopathy”. They also drew attention to the predominance of males and the age range of patients, concluding that “chagasic myocarditis is a disease of young individuals, disabling and killing them, mainly between 20 and 40 years old”^19^.

Ramos et al. published an article with a sequence of clinical studies in a region endemic for CD, in which two studies stood out for their approaches to CCC. In the first study evaluating 500 residents of the Cássia dos Coqueiros district in the State of São Paulo, they identified 39 cases of CCC, in which the ECGs showed a prevalence of 64.1% of right bundle branch blocks, followed by 30.7% of multifocal ventricular extrasystoles. In the second study of 72 patients with CCC, they again found a high prevalence of right bundle branch block (50% of cases), followed by extrasystoles (36%). Patients underwent multiple ECGs, and ECG mutability was observed in patients with CD. The authors concluded: “we could say that the electrocardiogram in chagasic myocarditis presents the most varied aspects and varies enormously within the same case”^20^. 

In a review of CD in the state of Minas Gerais, Pellegrino emphasized that “particular attention was given to the electrocardiographic method for the recognition and characterization of chronic ‘esquizotripanósica’ cardiopathy”. They indicated that the ECG pattern of CCC is not found in other cardiopathies or in individuals with negative serology for CD, reinforcing the fact that “around 75% of patients with chronic cardiopathy present with conduction disturbances, among which cases of right bundle branch block are the majority”^21^. 

Laranja et al. published a seminal work on CD in the Circulation Journal, presenting 1340 cases of acute or chronic CD. Regarding CCC, the authors recalled that “the diagnostic value and several peculiar features of the electrocardiographic findings in chronic Chagas' heart disease have been established based on an analysis of data from comparatively large groups of patients. Various authors have extensively confirmed these findings in studies of cases from different endemic areas". In a study of 683 patients with CCC, Laranja et al. described the main ECG changes, confirming a higher prevalence of conduction disturbances (55.9%), mainly represented by right bundle branch block (48.3%), followed by rhythm changes (47.9%) and, predominantly ventricular extrasystoles (42.6%). Most patients were male, and two-thirds were between 11 and 40 years of age, with a few patients above 50 years of age, reinforcing the young age of these individuals. This study represents the foremost contribution to the advancement of the understanding and global dissemination of knowledge regarding CD, with a particular focus on CCC^22^. 

## ELECTROCARDIOGRAM CONSIDERATIONS IN CHAGAS DISEASE

The 2009 Brazilian Society of Cardiology guidelines established a standard for the analysis and issuance of ECG reports^23^. It discusses the criteria for the technical evaluation of tracings related to ECG calibration, electrode placement, automated reports, and Internet reports. Prineas and Blackburn developed a classification scheme for population-based research and subsequent statistical analyses of coronary diseases. This method for ECG interpretation was the first suitable for application in clinical, epidemiological, cross-sectional, and longitudinal studies and became known as the Minnesota Code^24^.

## READING CODES

Three codes are usually used for ECG reading: the New York Heart Association (NYHA) nomenclature^25^, the adapted Minnesota Code^26^, and the Buenos Aires method^27^. 

The NYHA nomenclature does not include standardization criteria for performing ECGs. The definition of sinus bradycardia and complete or incomplete bundle branch blocks are inappropriate for CD. Regarding the adapted Minnesota Code, despite modifications in the assessment of arrhythmias and intraventricular conduction disturbances, some ECG aspects for evaluating coronary disease were retained, which do not apply to CCC, and the criteria for evaluating right or left ventricular hypertrophy in adults are incomplete. Although the Buenos Aires method is considered the most suitable for ECG reading in individuals with CCC, it does not evaluate the maximum normal limits for the PR interval based on the individual’s heart rate and age, and does not address the diagnosis of ventricular hypertrophy in the pediatric population.

In a review of reading codes in CD, Gonçalves and Prata concluded that in some studies, the Minnesota Code was used for ECG evaluation in CD. However, as it did not allow coding for complex arrhythmias and characteristic ventricular conduction disturbances, it was modified, creating the “adapted Minnesota Code”^28^. Several researchers have used this adapted code in their studies on CD^29^^,^^30^. Even with advancements in the use of the adapted Minnesota Code, some imperfections continue to hinder result comparisons. Despite the emergence of the adapted Minnesota Code, the NYHA nomenclature remained in use^4^^,^^31^^,^^32^, making the comparison of results in studies on CD even more difficult.

## STANDARDIZATION

Standardizing ECG interpretation is essential for facilitating the comparison of electrocardiograms across diverse studies and for appraising the outcomes of research using this diagnostic approach. Therefore, standardization should not be limited solely to the reading of the obtained tracing but also to the conditions and rules for conducting the examination^28^. The World Health Organization convened a group of experts on the feasibility of epidemiological and analytical studies on CD, which identified the need to standardize protocols for ECG interpretation and recommended the implementation of guidelines and ECG criteria for the diagnosis of CCC^33^. Following this recommendation, the Ministry of Health of Argentina provided the first document that systematized ECG alterations defining CCC^34^. Twenty years later, through the Brazilian Consensus on Chagas Disease, the Ministry of Health of Brazil standardized alterations compatible with CCC^35^. In the context of clinical trials, the BENEFIT study systematized alterations that are directly related to the progression of CCC^36^.

## REPRODUCIBILITY

Muynck and Romero evaluated the reproducibility of ECG changes in CCC and found that, on average, the intra-observer reproducibility was 85% and the inter-observer reproducibility was 75%. Among the electrocardiographic findings, the complete right bundle branch block showed the highest reproducibility in both observations: 100% intra-observer and 65% inter-observer. Their other findings showed limited reproducibility^37^. Lázzari et al., in an epidemiological study of CD, showed that intra-observer agreement (87-96%) was uniformly higher than inter-observer agreement (78-92%) when considering normal ECG vs. abnormal ECG. Although the interpretations of some ECG abnormality categories were highly reproducible, others, especially those with low frequencies, showed lower levels of agreement^27^.

## THE ELECTROCARDIOGRAM IN CHAGAS DISEASE

ECG changes are more prevalent in patients with CD than in those with negative *Trypanosoma cruzi* serology, and this has been demonstrated in many studies^22^. Almost all ECG abnormalities are found in CD, with a predominance of abnormalities in the formation and conduction of cardiac impulses^3^. In the presence of other types of heart diseases, electrocardiographic changes characteristic of these conditions can overlap with those typical of CCC; therefore, ECG does not constitute a method with high specificity for detecting CCC^3^.

The complex electrophysiological substrate generated by CCC lead to a wide variety of ECG manifestations. The classic findings, although not pathognomonic, comprise right bundle branch block, complete or incomplete, found in 13-48% of patients with CCC, and are often associated with left anterior hemiblock and ventricular extrasystoles^3^. Many of these alterations are due to an anatomical substrate of inflammation and fibrosis, initially predominantly affecting the conduction system, mainly the right bundle branch of the His bundle, extending to the left anterosuperior fascicle, and eventually becoming more diffuse and nonspecific^38^. This pattern makes the posterior-inferior fascicle block uncommon, as well as the left bundle branch block, the latter being at least 10 times less frequent than the right bundle branch block in this heart condition^39^. The duration of the QRS complex is directly related to the dimensions of the left ventricle and inversely related to the systolic function of the left ventricular, especially with a left bundle branch block^40^. Increased QRS duration is also an independent prognostic factor for left ventricular dysfunction in CCC^41^. The presence of ventricular ectopic activity is also very common, affecting 15-47% of individuals^3^. The higher the frequency and polymorphic features of the premature ventricular beat, especially if associated with other ECG changes, the stronger the relationship between the severity of CCC and left ventricular dysfunction and enlargement^42^. The presence of sustained and non-sustained ventricular tachycardia is an independent prognostic factor, especially for sudden death, with reports of this event being the first clinical manifestation of the disease^43^. Ventricular arrhythmia can be recorded in short routine tracings, but its paroxysmal and intermittent nature makes conventional ECG not ideal for its detection; 24 hour Holter monitoring is more commonly used^44^. Supraventricular ectopic activity is less common, with supraventricular extrasystoles occurring in 1.5-12%, without conferring prognostic value. Atrial fibrillation (AF) is the most common arrhythmia in clinical practice, and the association between the occurrence of AF and poor prognosis in patients with Chagas heart disease has been recognized since its seminal description by Carlos Chagas in 1922^11^. The reported prevalence of AF in patients with CD varies from 5.3-10% depending on the studied population^39^, typically associated with pronounced myocardial damage, diffuse involvement of the conduction system, ventricular arrhythmias, and ventricular dysfunction^39^. 

The atrioventricular conduction system is affected in a significant proportion of patients. The main ECG manifestations of sinoatrial dysfunction include sinus bradycardia, especially if the sinus rate is less than 50 bpm; sinus arrest; second-degree sinoatrial block; and escape rhythms denoting the inhibition of the normal sinus pacemaker, such as junctional and accelerated idioventricular rhythms. In the atrioventricular node, most atrioventricular blocks (AVBs) occur because of lesions distal to the His bundle trunk, including first-second degree AVB (Types I, II, 2:1, or advanced) and complete (third-degree) AVB. These alterations are found in up to 28% of cases with first- and second-degree AVB and up to 11% of complete AVBs^3^. First- and second-degree AVBs can be associated, among other ECG alterations, with intraventricular conduction disturbances. Concomitant with a complete right bundle branch block associated with a left anterior fascicular block, they generally indicate an advanced and diffuse conduction system injury and a higher likelihood of progression to a complete block^45^. Other significant alterations including pathological Q waves and peripheral low voltage, especially in advanced stages of CCC, appear to have an independent prognostic value but a borderline positive predictive value^43^. Repolarization changes occur in up to 40% of ECGs, and although there are reports of the isolated importance of repolarization changes, primary ventricular repolarization changes appear to be in an intermediate stage of involvement without a significant isolated value^3^. 

Few studies have addressed electrocardiographic criteria for defining CCC. Experts and researchers of CCC discussed these criteria and merged them into the first version of the Brazilian Consensus on Chagas Disease (2005)^2^. In a review, Biolo et al. classified electrocardiographic changes as non-specific and typical, defining CCC, according to the Brazilian consensual guidelines on CD^46^. Thus, the typical electrocardiographic alterations that define CCC are as follows: sinus bradycardia < 40 bpm; frequent ventricular extrasystoles (> 1); complete right bundle branch block, associated or not with left anterior hemiblock; primary alteration of ventricular repolarization; second to third degree atrioventricular block; complete left bundle branch block; electrically inactive zone; sinus node dysfunction; non-sustained ventricular tachycardia; atrial fibrillation; atrial flutter; and ventricular fibrillation. (Table 1)

**TABLE 1:** Electrocardiographic alterations in Chagas disease (adapted from Biolo et al.^46^.

Typical alteration	Non-specific alteration
Sinus Bradycardia ≤ 40 bpm	Sinus Bradycardia ≥ 40 bpm
Frequent Ventricular Premature Beats (> 1)	Isolated Ventricular Premature Beats
Complete Right Bundle Branch Block	Incomplete Right Bundle Branch Block
Primary Ventricular Repolarization Alteration	Secondary Ventricular Repolarization Alteration
2nd and 3rd Degree Atrioventricular Block	1st Degree Atrioventricular Block
Complete Left Bundle Branch Block	Incomplete Left Bundle Branch Block
Electrically Inactive Area	Left Axis Deviation
Sinus Node Dysfunction	Sinus Arrhythmia
Non-sustained Ventricular Tachycardia	Sinus Tachycardia
Atrial Fibrillation	Left Anterior Hemiblock
Atrial Flutter	Low Voltage
Ventricular Fibrillation	Migratory Pacemaker

## DISCUSSION

Baseline ECG is a low-cost, simple, and routine examination that provides clinical information for diagnosis and prognosis. However, the complex and heterogeneous nature of CD, with several ECG manifestations, makes it challenging to compare studies involving patients with CCC. Owing to the progressive nature of CD, ECG is used for both clinical monitoring and prognostic evaluation. Given the high mortality associated with CD^47^, it is essential to assess the risk through scores. The Rassi score is a useful tool for stratifying the risk of death in patients with CCC, with points assigned to simple characteristics in its construction and obtained through basic assessment methods, such as ECG^7^. This score allows the detection of significant strata in the risk of mortality in patients with CCC. Another study that categorized the risk of mortality formulated a predictive model to assess the likelihood of sudden death among individuals with CCC. This model identified four distinct predictive factors, two of which were alterations in the ECG: dispersion of the QT interval and ventricular extrasystoles^48^. 

In the context of clinical research, follow-up protocols for patients with CD include serial ECGs^36^. However, most of these studies did not follow a standardized pattern and use heterogeneous classification codes and criteria for ECG progression^49^. In a general sense, some studies consider non-specific ECG changes to be progressive, such as secondary alteration of ventricular repolarization, first-degree atrioventricular and intraventricular conduction disorders, isolated left anterior hemiblock, isolated supraventricular and ventricular extrasystoles, and left ventricular hypertrophy. However, these changes are included in defining CCC, and an overestimation in the diagnosis of CCC and patients at risk of developing heart failure will ensue.

Longitudinal studies that used ECG to assess progression from the indeterminate form to CCC do not always show in their method which ECG changes they considered as defining CCC. In a systematic review of the progression from the indeterminate form of CD, most of the analyzed studies did not explicitly state the ECG criteria used to identify disease progression^50^. Furthermore, some studies have included non-specific electrocardiographic changes in defining progression^4^^,^^31^^,^^51^^-^^53^.

Another significant aspect is related to the risk stratification of CD found in consensus classifications and guidelines^54^. According to the current classifications, the approach for individuals with CD and normal ECG findings is not uniform^2^^,^^55^^-^^58^. The Brazilian Consensus on Chagas Disease does not include the indeterminate form of CD (normal ECG) in the CCC classification and classifies patients with abnormal ECG and normal echocardiogram as “stage A”. The Latin American Guideline for the Diagnosis and Treatment of Chagas Cardiomyopathy includes the indeterminate form as “stage A” and patients with an abnormal ECG and normal echocardiogram as “stage B1” and was recently endorsed by the SBC Guideline on the Diagnosis and Treatment of Patients with Cardiomyopathy of Chagas Disease - 2023^59^. The modified Los Andes classification distinguishes patients with normal ECG and echocardiography findings from those with normal ECG and abnormal echocardiography findings. (Table 2) 

**TABLE 2:** Classifications of chronic Chagas heart disease and the ECG (adapted from Saraiva et al.^54^.

CCC classification	Staging	Normal ECG	Altered ECG
			
Modified Los Andes^55^ (1982)	IA / IB / II / III	IA / IB	II / III
Kuschnir^56^ (1985)	0 / I / II / III	0	I
Brazilian consensus^2^ (2005)	A / B1 / B2 / C / D	-	A / B1 / B2 / C / D
I Latin American guidelines^57^ (2011)	A / B1 / B2 / C / D	A	B1
American Heart Association Statement^58^ (2018)	A / B1 / B2 / C / D	A	B1

The stages used for each classification represent different survival expectations. Therefore, patients who are not comparable should not be included at the same stage^60^. Therefore, if the ECG criteria defining CCC are not used, there is a risk of including patients with the indeterminate form with non-specific ECG changes along with patients with ECG changes defining CCC^2^^,^^34^.

Electrocardiographic prognostic markers remain important targets for longitudinal studies^61^, and the standardization of ECG is essential for comparison studies using this method. Therefore, standardization of ECG interpretation, mainly the ECG criteria for CCC diagnosis, is crucial for both clinical research and practice in Chagas disease.

## CONFLICT OF INTEREST

The authors declare no conflicts of interest related to their association with the industry or personal financial support. They confirm their involvement in one review and five consensus or guideline projects addressing chagasic heart disease. The following is a compilation of these reviews and guidelines:

1. Brazilian Consensus on Chagas Disease (2005)
2. Latin American Guideline for the Diagnosis and Treatment of Chagasic Heart Disease (2011)
3. 2nd Brazilian Consensus on Chagas Disease (2015)
4. Multimodality imaging evaluation of Chagas disease: an expert consensus of the Brazilian Cardiovascular Imaging Department (DIC) and the European Association of Cardiovascular Imaging (EACVI) (2018)
5. Chagas heart disease: An overview of diagnosis, manifestations, treatment, and care (2020)
6. SBC Guideline on the Diagnosis and Treatment of Patients with Cardiomyopathy of Chagas Disease (2023)
